# Machine Learning of Bacterial Transcriptomes Reveals Responses Underlying Differential Antibiotic Susceptibility

**DOI:** 10.1128/mSphere.00443-21

**Published:** 2021-08-25

**Authors:** Anand V. Sastry, Nicholas Dillon, Amitesh Anand, Saugat Poudel, Ying Hefner, Sibei Xu, Richard Szubin, Adam M. Feist, Victor Nizet, Bernhard Palsson

**Affiliations:** a Department of Bioengineering, University of California—San Diego, La Jolla, California, USA; b Department of Pediatrics, University of California—San Diego, La Jolla, California, USA; c Skaggs School of Pharmacy and Pharmaceutical Sciences, University of California—San Diego, La Jolla, California, USA; d Department of Biological Sciences, University of Texas at Dallas, Richardson, Texas, USA; e Novo Nordisk Foundation Center for Biosustainability, Technical University of Denmark, Lyngby, Denmark; Hackensack Meridian Health Center for Discovery and Innovation

**Keywords:** RNA-seq, antibiotics, independent component analysis, iron regulation, machine learning, transcriptional regulation

## Abstract

*In vitro* antibiotic susceptibility testing often fails to accurately predict *in vivo* drug efficacies, in part due to differences in the molecular composition between standardized bacteriologic media and physiological environments within the body. Here, we investigate the interrelationship between antibiotic susceptibility and medium composition in Escherichia coli K-12 MG1655 as contextualized through machine learning of transcriptomics data. Application of independent component analysis, a signal separation algorithm, shows that complex phenotypic changes induced by environmental conditions or antibiotic treatment are directly traced to the action of a few key transcriptional regulators, including RpoS, Fur, and Fnr. Integrating machine learning results with biochemical knowledge of transcription factor activation reveals medium-dependent shifts in respiration and iron availability that drive differential antibiotic susceptibility. By extension, the data generation and data analytics workflow used here can interrogate the regulatory state of a pathogen under any measured condition and can be applied to any strain or organism for which sufficient transcriptomics data are available.

**IMPORTANCE** Antibiotic resistance is an imminent threat to global health. Patient treatment regimens are often selected based on results from standardized antibiotic susceptibility testing (AST) in the clinical microbiology lab, but these *in vitro* tests frequently misclassify drug effectiveness due to their poor resemblance to actual host conditions. Prior attempts to understand the combined effects of drugs and media on antibiotic efficacy have focused on physiological measurements but have not linked treatment outcomes to transcriptional responses on a systems level. Here, application of machine learning to transcriptomics data identified medium-dependent responses in key regulators of bacterial iron uptake and respiratory activity. The analytical workflow presented here is scalable to additional organisms and conditions and could be used to improve clinical AST by identifying the key regulatory factors dictating antibiotic susceptibility.

## INTRODUCTION

Antibiotic activities are highly dependent on environmental conditions. Nutrient availability, pH, host factors, and coadministered therapies can markedly alter antibiotic potency as calculated by MICs (1–6). Despite these dependencies, antibiotic susceptibility testing (AST) is performed under one standardized condition in nutrient-rich bacteriological media, most prominently cation-adjusted Mueller-Hinton broth (CA-MHB). CA-MHB poorly recapitulates *in vivo* conditions of the host, and prior studies have highlighted potential shortcomings of standardized AST in prioritizing treatment options against multidrug-resistant (MDR) pathogens (5–7). In many cases, AST performed in mammalian tissue culture media, such as RPMI 1640 or Dulbecco modified Eagle medium, may be a more accurate predictor of *in vivo* efficacy as it more closely mimics physiological conditions (5). However, current analytical methods are insufficient to reveal why cells respond differently to antibiotics across different medium types.

Systems analysis of omics measurements can elucidate global metabolic responses to antibiotic treatment (8–11). Previously, we published a high-quality transcriptomic compendium for Escherichia coli, named PRECISE, containing 278 expression profiles (12). Using independent component analysis (ICA), a machine learning method developed to separate mixed signals, we decomposed PRECISE into 92 independently modulated groups of genes, called iModulons. iModulons exhibit significant overlap with classically defined regulons, allowing for their direct association to one or more known transcriptional regulators. In addition, iModulons have been used to discover new regulons or to refine existing regulons by adding new genes and removing false regulatory interactions (12–15). This is an advantage over traditional gene set enrichment analysis, which requires predefined gene sets that may not be accurate. ICA simultaneously computes activities for each iModulon across every condition in the expression compendium, which predictably reflect the activity state of the enriched regulators.

iModulon activities can reveal the cellular state beyond the transcriptome. In one case, adaptive laboratory evolution of E. coli in excess iron conditions resulted in multiple mutations in the oxidative stress response regulator OxyR (16). In each of these evolved mutant strains, ICA revealed that the SOS-response regulator LexA was no longer activated by hydrogen peroxide exposure, indicating that the mutations reduced DNA damage from reactive oxygen species (ROS) through constitutive OxyR activation. In another study (17), iModulons were used to elucidate 5 major stress responses elicited when 42 different heterologous genes were expressed in E. coli. Each heterologous protein induced a specific iModulon response. iModulons have been successfully identified for additional species and applied to disentangle the transcriptional trajectory of a Bacillus subtilis sporulation time course (18) and identify medium-specific transcriptional responses in Staphylococcus aureus (19). Since iModulons often contain genes of unknown function, one can formulate hypotheses to discover these gene functions in different organisms (14, 15, 18). iModulons thus provide both mechanistic understanding and a guide to discovery.

Here, we demonstrate the utility of an ICA-based workflow to deconvolute transcriptomic responses to antibiotics in E. coli K-12 MG1655. Medium-dependent conditional antibiotic susceptibility profiles for E. coli were measured in (i) glucose M9 minimal medium (M9), a defined salt-based medium; (ii) cation-adjusted Mueller-Hinton broth (CA-MHB), the standardized rich medium for antibiotic susceptibility testing; and (iii) Roswell Park Memorial Institute medium (RPMI) 1640 with 10% Luria broth (LB; R10LB), a medium that mimics physiological conditions. ICA of expression profiles in these three media identified key transcriptomic differences in metabolism and stress responses. Further, ICA following exposure to subinhibitory concentrations of antibiotics found that such treatment decelerated E. coli respiration specific to rich bacteriological media. Our work highlights the metabolic and physiologic shifts that E. coli undergoes in response to various nutritional environments and upon antibiotic exposure, providing a roadmap for interrogating such effects in other bacterial species.

## RESULTS

### A few mechanisms can provoke large transcriptomic perturbations.

**FIG 1:**
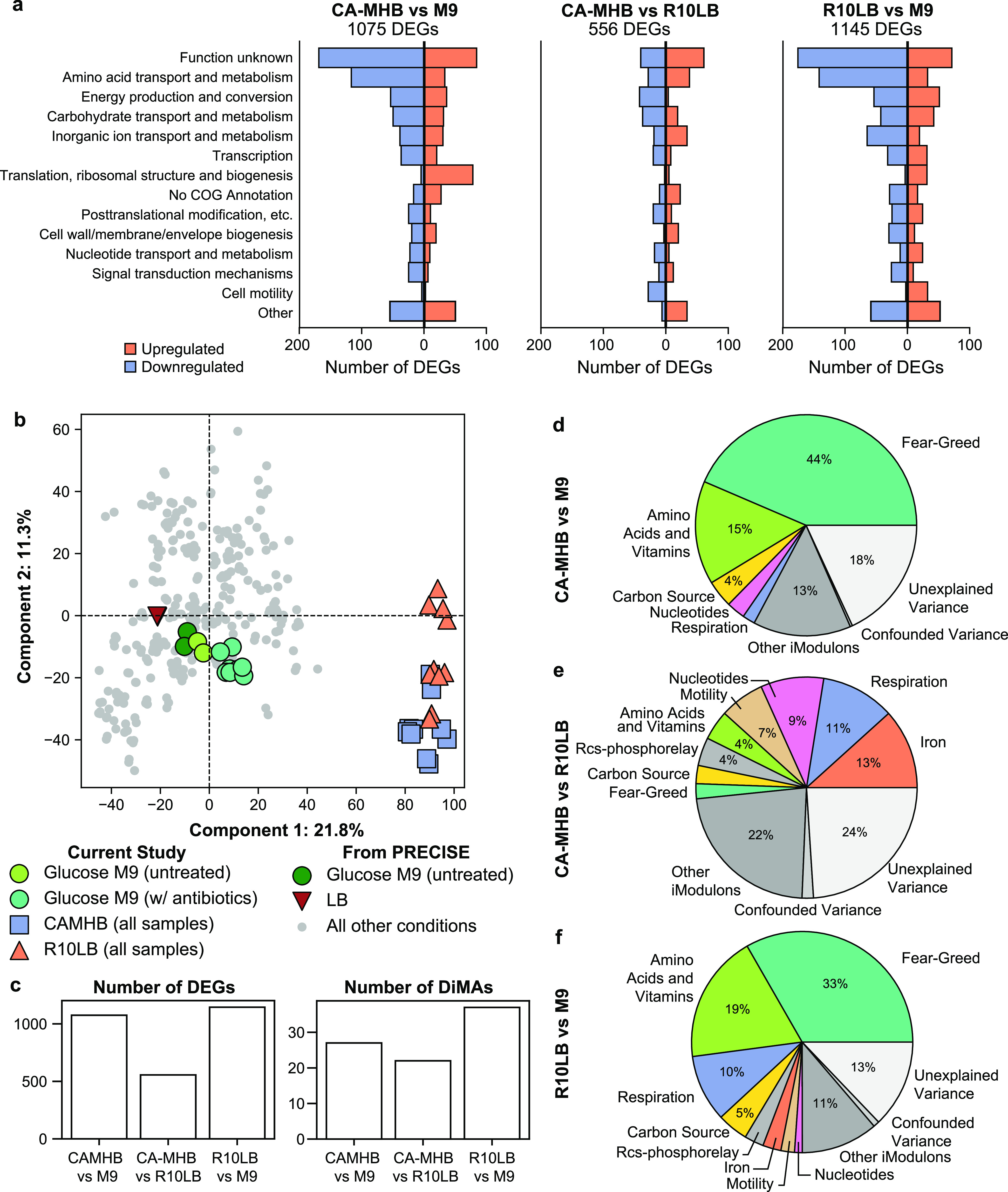
Global comparative analysis of the E. coli transcriptome across three media compositions. (a) Pairwise differentially expressed genes across COG categories between CA-MHB media, M9 minimal media, and R10LB media. Orange bars represent upregulation and blue bars represent downregulation. (b) Top two principal component loadings for the PRECISE data set, comprising 278 RNA-seq profiles (12), combined with the data generated in this study. (c) Number of differentially expressed genes (DEGs) compared to the number of differential iModulon activities (DiMAs). (d to f) Proportion of expression deviation explained by eight groups of iModulons. iModulon activities in each group are shown in Fig. 2 and Fig. S2b and d and reported in Table S1.

Prior studies revealed interrelationships between medium composition and bacterial antibiotic susceptibility by contrasting drug activity profiles under multiple medium conditions. To investigate the basis for medium-dependent antibiotic activities, we first defined the baseline effect of medium composition on E. coli K-12 MG1655 gene expression by performing RNA-seq during growth in CA-MHB, M9, or R10LB. We identified 1,075 differentially expressed genes (DEGs) between the physiological medium CA-MHB and nutrient-poor M9 minimal medium, comprising nearly one-quarter of the E. coli genome (Fig. 1a). These DEGs spanned every Cluster of Orthologous Groups (COG) category, with 24% of DEGs of unknown function. R10LB yielded transcriptional responses more similar to CA-MHB than M9, since only 556 genes were differentially expressed between the two media (Fig. 1a).

10.1128/mSphere.00443-21.3 FIG S2 Additional iModulons with significant differences between media. (a) Bar chart showing percentage of iModulon activity explained by growth rate, as computed by the *R*^2^ from linear regression. Only iModulons with an R^2^ (i.e., fraction explained activity) above 0.1 are shown. (b to d) iModulon activities of Rcs-phosphorelay-related iModulons (b), motility iModulons (c), and all other iModulons (d) with significant differences across the three media. Download FIG S2, PDF file, 0.3 MB. Copyright © 2021 Sastry et al. 2021 Sastry et al. https://creativecommons.org/licenses/by/4.0/ This content is distributed under the terms of the Creative Commons Attribution 4.0 International license.

10.1128/mSphere.00443-21.8 TABLE S1 Statistically significant changes in iModulon activity between medium compositions. Download Table S1, XLSX file, 0.02 MB. Copyright © 2021 Sastry et al. 2021 Sastry et al. https://creativecommons.org/licenses/by/4.0/ This content is distributed under the terms of the Creative Commons Attribution 4.0 International license.

For context, the RNA-seq data set generated in this study was compared to our previously published high-quality compendium containing 278 E. coli expression profiles, PRECISE (12), which primarily contains samples grown in M9 minimal medium or LB. Principal-component analysis (PCA) of the combined data set showed that CA-MHB and R10LB provoked significantly different responses from those probed in PRECISE, including growth on LB media without RPMI (Fig. 1b). As a control, gene expression in glucose M9 minimal medium was similar to expression profiles on identical medium produced previously (20) (Pearson *R* = 0.92), highlighting the internal consistency of the RNA-seq analysis. This earlier expression profile grown on glucose M9 minimal medium served as the reference condition, to which all further iModulon activities are compared.

We combined the 278 data sets in PRECISE with the 30 new RNA-seq data sets from this work to form a new compendium of 308 profiles (see the supplemental material). Application of ICA identified 98 iModulons, 88 of which were nearly identical to those extracted from the original PRECISE data set (12) (see Fig. S1). These iModulons are presented in iModulonDB (iModulonDB.org), an interactive visualization tool (21). To dissect the transcriptional differences between the three medium formulations, we computed the differential iModulon activities (DiMAs) between each pair of media. DiMAs are iModulons that have large, statistically significant (false discovery rate [FDR] > 0.1) activity changes between two conditions of interest. DiMAs are akin to coarse-grained DEGs but can be directly traced back to specific transcriptional regulators. In contrast to the hundreds of DEGs between the medium conditions, a maximum of 37 iModulons were perturbed between these conditions, simplifying further analysis by an order of magnitude (Fig. 1c).

10.1128/mSphere.00443-21.2 FIG S1 Comparison of iModulons after adding new data to PRECISE. Venn diagram comparing iModulons generated from PRECISE compared to iModulons generated from PRECISE + 30 new expression profiles. Four iModulons from the original PRECISE dataset were not found upon addition of new data, likely because they were initially weaker signals. Download FIG S1, PDF file, 0.1 MB. Copyright © 2021 Sastry et al. 2021 Sastry et al. https://creativecommons.org/licenses/by/4.0/ This content is distributed under the terms of the Creative Commons Attribution 4.0 International license.

On average, iModulons could explain 81% of the difference in expression between each pair of medium conditions. To further coarse grain our analysis, we grouped iModulons together based on regulator function (see Table S1 in the supplemental material). Borrowing from multiple linear regression, we calculated how much of the expression deviation could be directly explained by each iModulon and each iModulon group (Fig. 1d to f). The single largest contributor to expression variation between CA-MHB and M9 was the RpoS iModulon, which accounted for 30% of the variation in expression. Conversely, the top contributors to expression variation between CA-MHB and R10LB were the Fur-1, PurR-2, FlhDC, NarL, and Fnr iModulons. In total, these five iModulons explained 34% of the variance between CA-MHB and R10LB (see Table S2).

10.1128/mSphere.00443-21.9 TABLE S2 Fraction of expression variance explained by each iModulon between medium compositions. Download Table S2, XLSX file, 0.01 MB. Copyright © 2021 Sastry et al. 2021 Sastry et al. https://creativecommons.org/licenses/by/4.0/ This content is distributed under the terms of the Creative Commons Attribution 4.0 International license.

### iModulons elucidate transcriptional markers of “greedy” growth in rich media.

**FIG 2:**
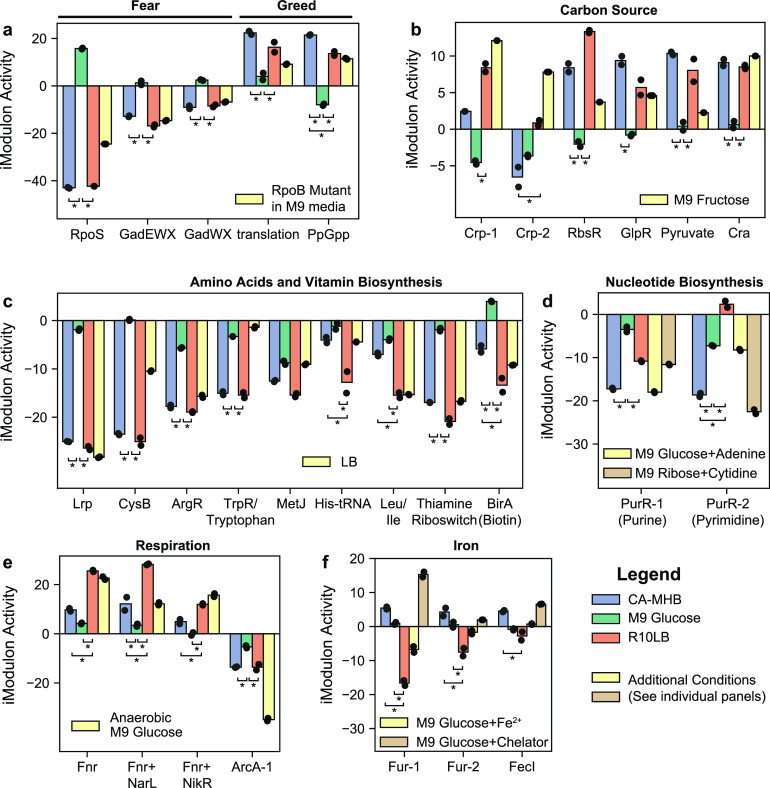
Mechanisms underlying the complex transcriptional response to different media. Bar charts show the iModulon activities for fear-greed iModulons (a), carbon source catabolism iModulons (b), amino acid and vitamin B iModulons (c), nucleotide biosynthesis iModulons (d), respiration iModulons (e), and iron-related iModulons (f). iModulon activities are computed relative to a reference condition (wild-type MG1655 grown in M9 minimal media from the PRECISE database). Individual measurements for independent biological replicates are plotted on top of bars. Asterisks (*) indicate a statistically significant differential iModulon activity (FDR < 0.1) between two of the three medium compositions (CA-MHB, M9, and R10LB). All other iModulons with significant activity differences are shown in Fig. S2b to d.

The major source of expression variation between rich media (CA-MHB and R10LB) and minimal medium (M9) could be explained by the “fear-greed” trade-off, which balances fast growth (i.e., “greed”) and high stress response readiness (i.e., “fear”) (12, 22) (Fig. 2a). In rich media, bacterial cells experience significantly less environmental stress, leading to a strong decrease in the activity level of stress-related iModulons, such as RpoS and the GadEWX acid-response system (23). This reduced stress in turn frees cellular resources that can be allocated to promote growth, such as increased activity levels of iModulons encoding genes related to protein translation. These two processes are molecularly linked through (p)ppGpp, a signaling molecule that is produced when the cell experiences nutrient limitations or other stresses. ppGpp binds to RNA polymerase, changing its conformation to activate genes under the control of the RpoS stress response sigma factor and decrease transcription of genes related to translation (24, 25).

A mutated RNA polymerase (RpoBE672K) observed during adaptive laboratory evolution on glucose minimal media (22) is hypothesized to interfere with the ppGpp binding site on RNA polymerase. The iModulon activities of the WT strain under rich media are similar to the iModulon activities of the RNA polymerase mutant strain; both exhibit increased expression of the genes in the translation and ppGpp iModulons and decreased expression of genes in the stress-related RpoS and GadEWX iModulons. It is important to note that the direction of iModulon activities changes reflect the expression changes in iModulon genes (i.e., both derepression and binding of activators results in higher iModulon activities). Overall, these activities indicate that, as expected, minimal media induce more (p)ppGpp stress alarmone production than rich media.

Growth rate-dependent changes in gene expression have been observed to potentiate antibiotic resistance and tolerance (26). Using the growth rate information from PRECISE, we found that growth rate effects best explained the RpoS and translation iModulon activities (*R*^2^ = 0.39 and 0.30, respectively). However, growth effects on biosynthesis and energy metabolism iModulon activities were more muted (see Fig. S2a), indicating that the fear-greed iModulons are the primary targets of growth rate-dependent expression changes.

Many iModulons related to carbon uptake were differentially activated between the three growth media, including those related to the global regulators CRP and Cra (Fig. 2b). CRP represses secondary carbon sources in the presence of glucose and is activated by cAMP, whereas Cra represses glycolysis and is deactivated by fructose derivatives (20). iModulons regulating alternative carbon sources, such as ribose (RbsR), glycerol (GlpR), and pyruvate, were active in both CA-MHB and R10LB, even though glucose is a component of RPMI. The Cra iModulon contains many glycolytic genes and was active in both RPMI and CA-MHB. iModulon activities for M9 media with fructose as a carbon source are shown in Fig. 2b, since many carbon uptake iModulon activities for this condition were similar to CA-MHB and R10LB.

Biosynthetic pathways for vitamins and amino acids contributed to 15 and 19% of the expression deviation from M9 for CA-MHB and R10LB, respectively (Fig. 2c). Since R10LB contains 10% LB, we included the LB activities for comparison. The iModulon activities of amino acid and B-vitamin iModulons are quite similar in CA-MHB, R10LB, and LB, suggesting a similar nutritional content. The main deviations between CA-MHB and R10LB appear to be in histidine and branched-chain amino acid biosynthesis that are regulated by tRNA-mediated transcriptional attenuation.

The PurR-2 iModulon, which controls pyrimidine biosynthesis, exhibited significantly lower activities in CA-MHB than in R10LB. Lower PurR iModulon activities are associated with nucleotide supplementation, indicating higher pyrimidine content in CA-MHB than in R10LB (Fig. 2d). On the other hand, *de novo* purine biosynthesis pathway was downregulated in both CA-MHB and R10LB relative to M9 medium.

iModulons related to anaerobic respiration were another large contributor to expression deviation in CA-MHB compared to the other two media (Fig. 2e). Three iModulons were enriched with the transcription factor Fnr: one iModulon uniquely enriched with the Fnr regulon, one iModulon enriched with genes coregulated by Fnr and the nitrate-responsive regulator NarL, and a final iModulon containing all genes coregulated by Fnr, NarL, and the nickel regulator NikR. An additional iModulon in this group was related to ArcAB, a two-component system that senses the redox state of quinone electron carriers to regulate the tricarboxylic acid (TCA) cycle among other genes (27, 28). ArcA iModulon activities were partially reduced in both CA-MHB and R10LB.

Iron regulation accounted for a large portion of the expression deviation between CA-MHB and R10LB, as represented by two Fur-enriched iModulons and the FecI iModulon (Fig. 2f). Fur binds to free iron (FeII) and represses genes related to iron siderophore synthesis and transport (29). Low iModulon activity, as seen in R10LB, indicates repression of Fur-regulated genes. FecI senses iron (III) and activates the expression of iron (III) uptake genes.

A few additional iModulons were differentially activated across the multiple medium conditions, and coincidently discriminate the biological processes that are perturbed between the reference M9 expression profile in PRECISE and the expression profiles from M9 media generated in this study. Three iModulons related to the Rcs-phosphorelay were activated in the new M9 and CA-MHB expression profiles (see Fig. S2b). The FliA iModulon, which controls chemotaxis, was activated in all three media, whereas the FlhDC iModulon, which controls biosynthesis and assembly of flagella, was only activated in R10LB (see Fig. S2c).

Although the three media induced vastly different gene expression changes, we used iModulons to simplify the analysis from hundreds of differentially expressed genes to a few dozen key regulators. This interpretation provided a tractable understanding of how medium components affect transcriptional regulation and laid the groundwork for investigating responses to antibiotic treatment.

### Antibiotic activities vary across medium compositions.

To examine how bacterial transcriptional responses impact antibiotic susceptibility, we conducted standardized AST for several antibiotics against E. coli MG1655 in CA-MHB, M9, and R10LB (Table 1). Briefly, E. coli was grown overnight in each of the three media, subcultured the following morning, and grown until reaching the logarithmic phase (∼0.4 optical density at 600 nm [OD_600_]). Logarithmic phase cultures were diluted to an OD_600_ of 0.002 and exposed to serial dilutions of the nine selected antibiotics (see Materials and Methods) (6). After 20 h, the cell density was measured to identify the amount of drug required to inhibit ≥90% of the growth of the untreated controls. In three cases, cell growth was uninhibited in all drug concentrations (e.g., ampicillin [AMP] on M9); in one case, the MIC could not be determined (rifampin [RIF] on R10LB).

**TABLE 1:** MIC_90_ of nine antibiotics on three media

Antibiotic (abbreviation)^*a*^	Drug information		MIC_90_ (μg/ml)		
	Class	Target process	CA-MHB	M9	R10LB
Ampicillin (AMP)	Penicillin	Cell wall	64	>16	256
Ceftriaxone (CTR)*	Cephalosporin	Cell wall	0.25	0.016	0.25
Ciprofloxacin (CIP)*	Quinolone	DNA gyrase	0.016	0.008	0.004
Fosfomycin (FOS)	Fosfomycin	Cell wall	16	64	>64
Meropenem (MEM)*	Carbapenem	Cell wall	0.016	0.031	0.13
Nitrofurantoin (NIT)	Furan	Multiple	256	128	>512
Plazomicin (PLZ)	Aminoglycoside	30S inhibitor	0.25	0.13	0.13
Rifampin (RIF)	Rifampin	Transcription	16	8	NA
Trimethoprim-sulfamethoxazole (T/S)*	Pyrimidine/sulfa	Folate synthesis	1	0.13	1

a *, Selected for further analysis.

Based on clear medium-dependent alterations in activity, we selected four antibiotics for further investigation. Two selected antibiotics, ceftriaxone (CTR) and trimethoprim-sulfamethoxazole (T/S), had identical susceptibilities in the rich media (CA-MHB and R10LB) and a lower MIC_90_ in M9. Ciprofloxacin was selected as it was most effective in R10LB and least active in CA-MHB. Meropenem (MEM) resulted in the opposite trend in susceptibility, where it was most effective in CA-MHB and least active in R10LB.

**FIG 3:**
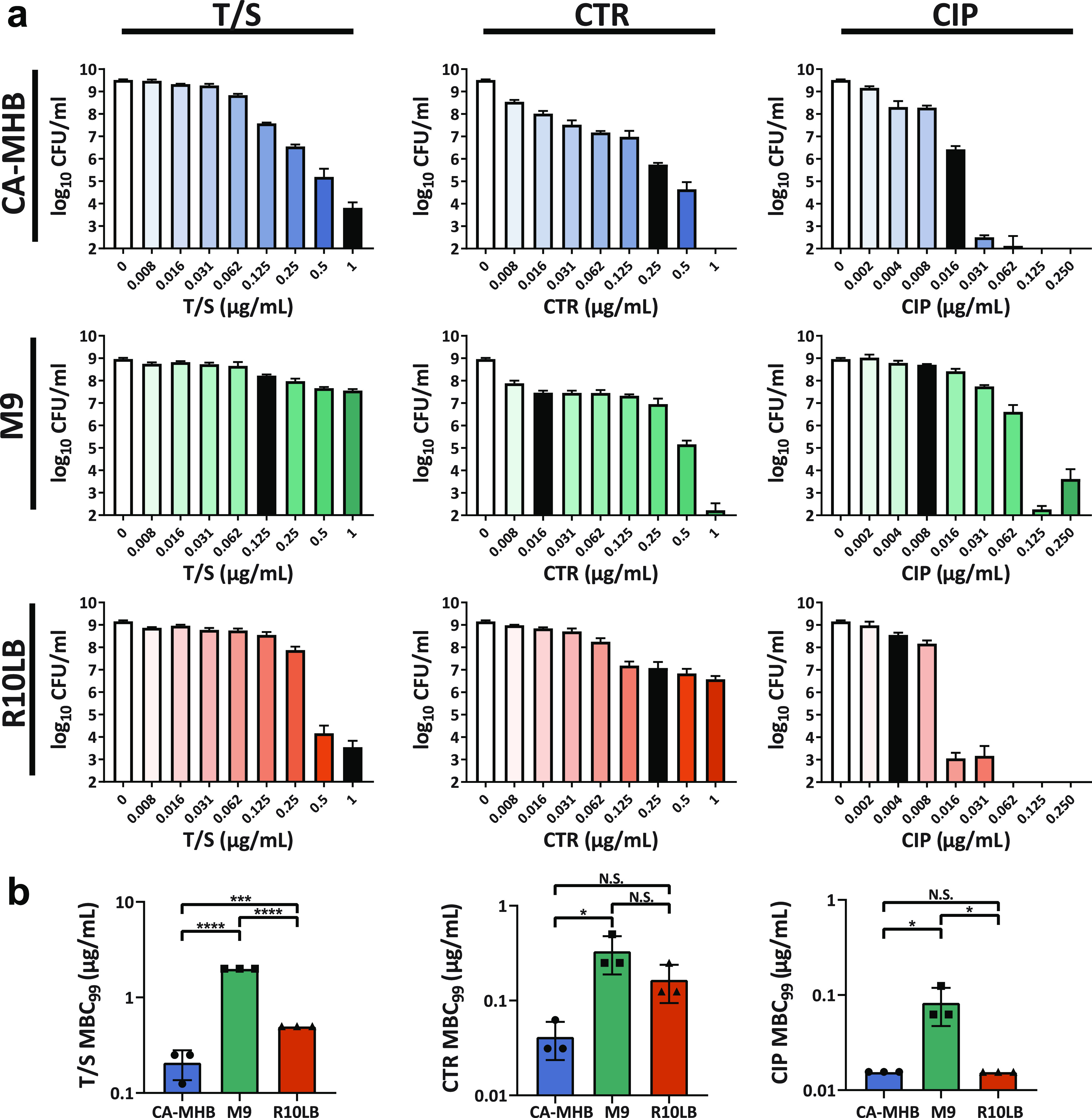
Minimum bactericidal concentration (MBC) of three antibiotics across three medium conditions. (a) Survival rates under different antibiotic concentrations. Black bars designate the concentration of antibiotics used in RNA-seq cultures (i.e., MIC_90_ in relevant media). Missing bars indicate the measured CFU are below the limit of detection. (b) Bar chart comparing MBC_99_ of each antibiotic across media. T/S, trimethoprim-sulfamethoxazole; CTR, ceftriaxone; CIP, ciprofloxacin.

According to the standardized AST as set forth by the Clinical and Laboratory Standards Institute (CLSI) (30), MIC_90_ was measured using an inoculum density of ∼1 × 10^5^ CFU/ml (Table 1). However, we required a significantly higher cell density (∼2.5 × 10^7^ CFU/ml) to collect enough mRNA for transcriptomic analysis. Since antibiotic susceptibility can be density dependent, we reassessed antibiotic bactericidal activity at the same culture density used for the mRNA isolations (Fig. 3a). From these data, we determined the minimum bactericidal concentration (MBC_99_) for each antibiotic in each medium type (Fig. 3b). At the higher culture densities, no differences were observed for ciprofloxacin (CIP) and CTR bactericidal activities in CA-MHB or R10LB, whereas T/S was more effective in CA-MHB than in R10LB. CIP, CTR, and T/S had the lowest bactericidal activity in M9. Despite being classified as bactericidal, no tested dose of MEM was able to eradicate MG1655 at these higher culture densities (see Fig. S3a). Corroborating the MBC_99_ results, MICs at the higher densities used for the RNA-seq analysis were likewise increased ∼5-fold from baseline MICs (see Fig. S3b).

10.1128/mSphere.00443-21.4 FIG S3 MIC and MBC for Meropenem. (a) Survival rates under different MEM concentrations. Black bars designate the concentration of MEM used in RNA-seq cultures (i.e., MIC_90_ in relevant media). (b) Fold change in MIC_90_ between a starting OD_600_ of ∼0.0004 versus 0.05. Download FIG S3, PDF file, 0.3 MB. Copyright © 2021 Sastry et al. 2021 Sastry et al. https://creativecommons.org/licenses/by/4.0/ This content is distributed under the terms of the Creative Commons Attribution 4.0 International license.

### Consistent transcriptional effects of subinhibitory antibiotic treatment.

Since the ICA-based decomposition of transcriptomes provided a clear explanation of the complex responses to different medium compositions, we applied a similar approach to investigate the effects of antibiotic treatment. The MIC_90_ of each antibiotic in each medium was selected as the experimental treatment dose, as indicated by the black bars in Fig. 3a. Cells were treated with antibiotics during exponential growth and sampled for RNA sequencing 30 min after treatment.

Gene expression after antibiotic treatment primarily clustered by medium, even when controlling for medium-specific responses (see Fig. S4a and b). Although MEM treatment did not kill MG1655 at any concentration (see Fig. S3a), it still induced small changes in the transcriptome (see Fig. S4c). However, these effects were not reflected in the MBC_99_, and we removed MEM-treated transcriptomes from subsequent analyses.

10.1128/mSphere.00443-21.5 FIG S4 Medium composition is the major determinant of gene expression. (a) Heatmap of gene expression values in log_2_-transformed transcripts per million (log-TPM) with hierarchical clustering. (b) Heatmap of expression fold changes of antibiotic-treated cells compared against the untreated control for the respective medium composition. (c) Significantly changed iModulon activities resulting from MEM treatment in M9 and CA-MHB media. No significant iModulons were detected in R10LB. Download FIG S4, PDF file, 0.6 MB. Copyright © 2021 Sastry et al. 2021 Sastry et al. https://creativecommons.org/licenses/by/4.0/ This content is distributed under the terms of the Creative Commons Attribution 4.0 International license.

**FIG 4:**
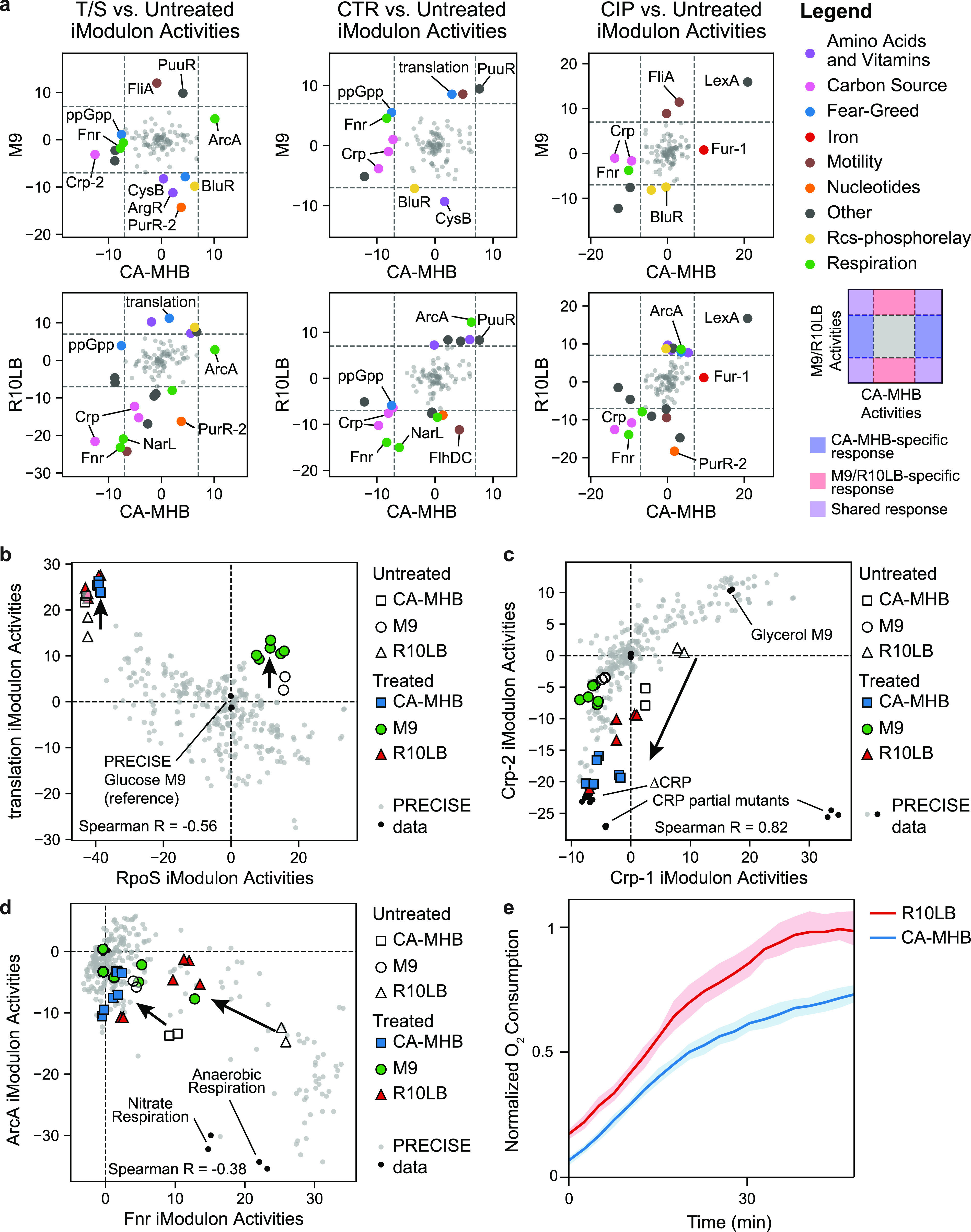
Selected transcriptional trade-offs that are independent of drug targets. Arrows indicate the effect of antibiotic treatment in rich media. (a) Pairwise comparison of iModulon activity changes between treated and untreated cells across three media. Colored points outside the dashed lines indicate statistically significant iModulon activities. (b) Scatterplot of the RpoS and Translation iModulon activities across PRECISE. (c) Scatterplot of the Crp-1 and Crp-2 iModulon activities across PRECISE. iModulon activities from CRP knockout and partial knockout strains are also shown. (d) Scatterplot of Fnr and ArcA iModulon activities across PRECISE. Anaerobic respiration conditions from PRECISE are also highlighted. (e) Oxygen consumption over time, as measured by normalized fluorescence, in CA-MHB and R10LB.

We first compared medium-specific changes in iModulon activity between antibiotic treatment versus untreated control in each of the three growth media (Fig. 4a and Table S3). Regardless of their mechanisms of action, several trends in iModulon activities were observed across all three antibiotics. Antibiotic treatment increased activity of the translation iModulon in M9 and R10LB (Fig. 4b) but had no consistent effect on the RpoS iModulon across the three antibiotic classes. Although none of the selected antibiotics interact directly with the ribosome, this indicated that antibiotic-mediated stress could indirectly increase ribosome levels in the cell.

10.1128/mSphere.00443-21.10 TABLE S3 Statistically significant changes in iModulon activity upon antibiotic treatment for all four antibiotics across the three medium types. Download Table S3, XLSX file, 0.01 MB. Copyright © 2021 Sastry et al. 2021 Sastry et al. https://creativecommons.org/licenses/by/4.0/ This content is distributed under the terms of the Creative Commons Attribution 4.0 International license.

We also observed large reductions in both Crp-related iModulon activities upon antibiotic treatment in rich media (Fig. 4c). These iModulons encode genes in secondary carbon source catabolism; the Crp-1 iModulon contains scavenging enzymes such as tryptophanase and acetyl coenzyme A synthetase, whereas the Crp-2 iModulon represents expression of phosphotransferase systems for alternative carbon sources. Lower activity of Crp iModulons indicated a reallocation of resources away from secondary carbon source catabolism. Traditional gene set enrichment analysis of the DEGs induced by antibiotic treatment could not identify Crp as a key regulator of the process, likely due to the size and breadth of the Crp regulon.

Across PRECISE (12), we observed a strong negative correlation between the iModulon activities of two major respiration-related transcription factors, Fnr and ArcA (Pearson *R* = −0.74, *P* < 10^−10^; Fig. 4d). In both CA-MHB and R10LB, antibiotic treatment resulted in significant drops in Fnr iModulon activities, coupled with a simultaneous increase in ArcA-regulated gene expression in the TCA cycle. Fnr contains an iron-sulfur cluster that is destabilized in the presence of molecular oxygen (31), rendering Fnr inactive. We hypothesized that the high expression of these genes in R10LB indicated a high respiration rate that left little intracellular molecular oxygen available to destabilize Fnr’s iron-sulfur cluster. To test this hypothesis, we measured oxygen consumption rates in CA-MHB and R10LB using an assay that is based on the ability of oxygen to quench the excited state of extracellular O_2_ consumption reagent (Fig. 4e). The normalized fluorescence is proportional to the depletion of oxygen concentration in the media (32, 33). Here lies one of the major differences between the two media; R10LB promotes a higher respiration rate than CA-MHB before antibiotic treatment.

Together, these two iModulons indicate that antibiotic treatment decelerated the high respiratory rate facilitated by R10LB. This process likely also reduced the redox state, as sensed by the ArcAB response system (27). A prior study used biochemical assays to investigate the effects of bacteriostatic antibiotics on E. coli (34) and observed that antibiotic treatment drastically reduced the respiration rate and moderately reduced the redox state of the cell. The drop in respiration was absent in minimal media, where we inferred a lower initial cellular respiration rate from the Fnr and ArcA iModulon activities.

Treatment of E. coli with three distinct antibiotics induced multiple common transcriptional changes, including (i) an increase in ribosomal gene expression, (ii) downregulation of secondary carbon catabolism, and (iii) a decelerated respiration rate in R10LB and CA-MHB. In the next section, we discuss transcriptional changes unique to each antibiotic.

### Specific effects of antibiotic treatment under different media.

In addition to the medium-specific iModulon activity trade-offs described above, each antibiotic induced specific changes in iModulon activity that could be correlated with differential antibiotic susceptibility.

**FIG 5:**
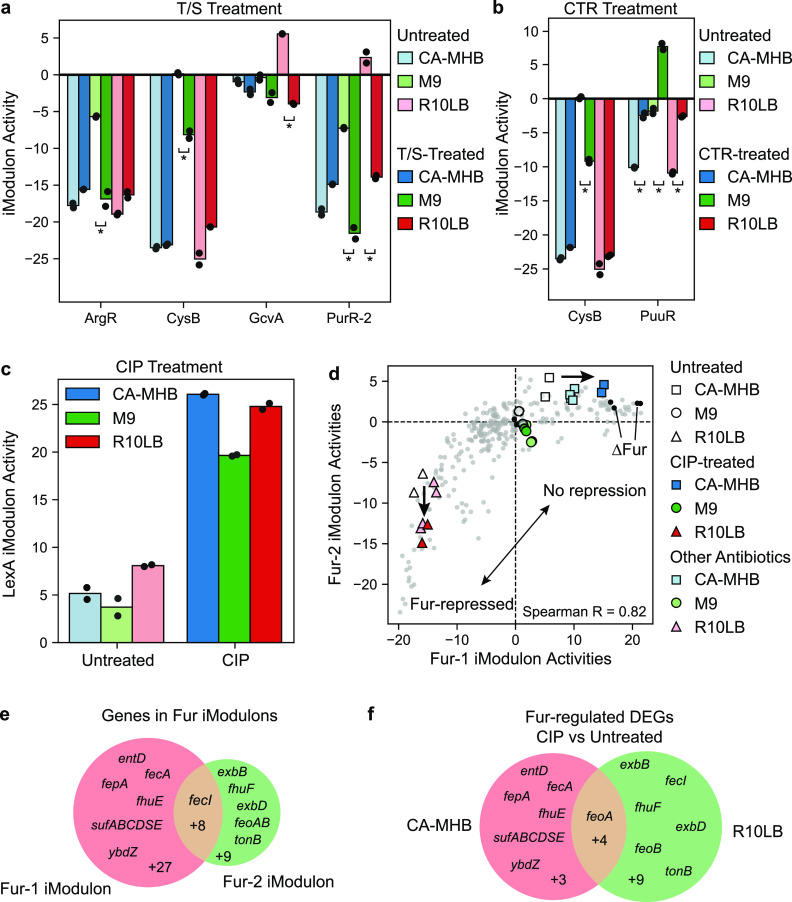
iModulon responses specific to antibiotic treatments. (a) iModulon activities of untreated and T/S-treated cells for four selected iModulons. Asterisks (*) represent statistically significant iModulon activity difference (FDR < 0.1). (b) iModulon activities of untreated and CTR-treated cells for two selected iModulons. Asterisks (*) represent statistically significant iModulon activity difference (FDR < 0.1). (c) iModulon activities of the DNA-damage response regulator LexA in untreated and CIP-treated cells. (d) Scatterplot of the Fur-1 and Fur-2 iModulon activities. Expression profiles from PRECISE are shown in light gray, and other colors are described in the legend. (e) Venn diagram comparing the genes in the Fur-1 and Fur-2 iModulons. (f) Venn diagram comparing the DEGs between ciprofloxacin and untreated cells in CA-MHB and R10LB media.

T/S is a combination of two drugs, both of which inhibit folate synthesis. E. coli was most susceptible to T/S on M9 media and less sensitive on R10LB and CA-MHB. Folate is used to synthesize purines, thymidylate, and methionine (35). However, the purine biosynthesis (PurR-1) and methionine biosynthesis (MetJ) iModulons were mostly unaffected by T/S treatment in any medium type, indicating that E. coli is not transcriptionally altering folate usage in response to T/S inhibition (see Fig. S5). Instead, we observed significant iModulon activity changes in four other amino acid and nucleotide-related iModulons (Fig. 5a). Sulfate utilization (CysB) and arginine biosynthesis (ArgR) iModulon activities decreased after treatment with T/S in M9 media. The glycine cleavage system (GcvA iModulon), which converts excess glycine to serine, was downregulated after treatment in R10LB, and pyrimidine biosynthesis (PurR-2) was downregulated after treatment in both M9 and R10LB. Although the MIC_90_ of T/S was identical in CA-MHB and R10LB, the underlying transcriptional responses to the aforementioned iModulons were different, indicating that these responses are not directly linked to T/S susceptibility.

10.1128/mSphere.00443-21.6 FIG S5 iModulon activities of untreated and T/S treated cells for iModulons requiring folate. No iModulon activities were significantly perturbed upon T/S treatment. Download FIG S5, PDF file, 0.2 MB. Copyright © 2021 Sastry et al. 2021 Sastry et al. https://creativecommons.org/licenses/by/4.0/ This content is distributed under the terms of the Creative Commons Attribution 4.0 International license.

The MIC_90_ of CTR for E. coli was also identical between CA-MHB and R10LB and higher for M9. Treatment with CTR in M9 media induced a medium-specific decrease in CysB iModulon activity (Fig. 5b). However, the PuuR iModulon increased after CTR treatment in all three media types, indicating a medium-independent response to CTR. PuuR responds to the polyamine putrescine and regulates its utilization and transport (36). Polyamine accumulation from sublethal antibiotic treatment has been observed previously and is hypothesized to reduce porin permeability to antibiotics (37).

The MIC_90_ of CIP was highest on CA-MHB and lowest on R10LB. CIP blocks DNA gyrase, creating double-stranded DNA breaks (38). Subsequently, after adding CIP to the culture, we observed a strong activity increase in the SOS-response regulator LexA iModulon in all three media indicating a media-independent response to CIP (Fig. 5c). CIP treatment also caused medium-specific effects on the two Fur iModulons (Fig. 5d and e; see also Fig. S6). The Fur-1 iModulon responds to iron starvation, derepressing iron (II) and (III) siderophore synthesis and transport systems, ribonucleotide reductases, superoxide dismutase, and iron-sulfur cluster assembly. The Fur-2 iModulon responds to excess iron, further repressing siderophore transport and repressing the energy-transducing Ton system. Together, these two iModulons trace out a trajectory from iron replete (Fur-repressed) to iron-poor (no Fur repression) conditions. CIP treatment in CA-MHB results in lower Fur-2 iModulon activities, indicating excess free iron availability. On the other hand, CIP treatment in R10LB, results in higher Fur-1 iModulon activities, indicating iron starvation. The difference in Fur iModulon activities may contribute to the differential susceptibility to CIP across the three media.

10.1128/mSphere.00443-21.7 FIG S6 Comparison of genes in the Fur-1 and Fur-2 iModulons. Genes grouped together are in the same operon (i.e., *feoAB* represents the operon containing *feoA* and *feoB*). Download FIG S6, PDF file, 0.2 MB. Copyright © 2021 Sastry et al. 2021 Sastry et al. https://creativecommons.org/licenses/by/4.0/ This content is distributed under the terms of the Creative Commons Attribution 4.0 International license.

The difference between the Fur-1 and Fur-2 iModulons are reflected in the DEGs between CIP-treated and untreated cells (Fig. 5f). In CA-MHB, CIP treatment increases the activity of the Fur-1 iModulon, and most of the respective DEGs are specific to the Fur-1 iModulon. On the other hand, CIP treatment in R10LB decreases the Fur-2 iModulon activity, resulting in DEGs specific to the Fur-2 iModulon. Due to the two dimensions of the Fur regulon, traditional gene set enrichment analysis of the DEGs does not identify Fur as a statistically significant enriched regulator (FDR < 0.01) in either medium.

## DISCUSSION

Bacterial responses to antibiotics are complex and depend on a variety of environmental and genetic factors. Here, we explored the effects of different medium compositions and antibiotic treatments on the transcriptomic state of the model strain E. coli K-12 MG1655. First, we showed that iModulons simplify the interpretation of large changes in expression due to various environmental conditions. Second, using iModulons, we identified environmental factors that contribute to the observed differences in the medium dependency potency of specific antibiotics against E. coli.

By combining iModulon activities with current knowledge of transcriptional regulators, we inferred that E. coli grown in R10LB have a higher respiration rate than those grown on CA-MHB. This observation was validated by direct oxygen consumption measurements. This shift would likely be missed using traditional differential expression analysis, since Fnr has a complex relationship with other respiratory regulators (28). However, the Fnr iModulon contained genes regulated specifically by Fnr and summarized their expression changes as its iModulon activity. Thus, iModulons expand upon the insight gained from simple differential gene expression.

A previous study also identified a connection between antibiotic treatment and respiration (34). It was observed that bacteriostatic antibiotics suppressed cell respiration, whereas cell death from bactericidal antibiotics was accompanied with accelerated respiration. The three antibiotics used in the present study (T/S, CTR, and CIP) have bactericidal effects, but we observed a deceleration in respiration under rich media (R10LB and CA-MHB). Two factors may account for the observational differences between the two studies: (i) the prior study grew cells in supplemented M9 minimal media, and (ii) the present study used subinhibitory concentrations of antibiotics, whereas the prior study used concentrations at 10× MIC. Further research is required to elucidate the interaction between respiration and cell killing from bactericidal antibiotics in nutrient-rich and nutrient-poor medium compositions.

We also observed a significant difference in Fur activity between bacteriological media (CA-MHB) and physiological media (R10LB), yielding a hypothesis to explain the differential susceptibility to ciprofloxacin in the two media. The Fur iModulon activities diverge further upon CIP treatment, indicating an increase of free iron in R10LB and a stronger iron starvation response in CA-MHB. This divergence is an especially surprising finding as iron is more abundant (∼330 μM) in CA-MHB compared to R10LB (∼60 μM) (39–41), and prior studies with E. coli have found an accumulation of iron in bacteriologic medium versus minimal media in the absence of antibiotic treatment (42). Between the observed increased intracellular oxygen levels and the increase in free iron specific to R10LB, increased susceptibility of E. coli to CIP in R10LB may be related to altered levels of ROS.

ROS, such as those created by Fenton chemistry, may contribute to the antibacterial action of CIP (43–45). In agreement with our findings, prior studies have also linked the activity of levofloxacin to iron accumulation and oxidative stress in Streptococcus pneumoniae (46), suggesting fluoroquinolone-mediated alterations in the Fur iModulon may be the basis for the bacterial accumulation of iron and subsequent increased sensitivity to CIP. Iron overload has been proposed as a parallel pathway to mediate evolutions of antibiotic resistance to ciprofloxacin in E. coli (47). However, in light of the controversy regarding the role of oxidative stress in antibiotic lethality (48–50), further studies investigating this relationship are warranted.

The two-dimensional representation of the Fur regulon indicates that expression changes are not consistent across the entire Fur regulon. Under iron starvation, genes in the Fur-1 iModulon respond strongly to changes in Fur activity, whereas under iron-replete conditions, genes in the Fur-2 iModulon respond more to changes in Fur activity. The plasticity of the Fur regulon has been an open question, since previous studies found that changes in Fur activity can result in different sets of DEGs depending on the initial cellular state (47, 51). This example highlights the power and sensitivity of iModulon-based analysis compared to traditional analyses.

Although the susceptibility of E. coli to T/S and CTR were identical on CA-MHB and R10LB, MIC_90_ measurements do not capture the full story. The activities of multiple iModulons, including GcvA, Fnr, and Crp, were altered by antibiotic treatment in different ways between the two media. The folate synthesis inhibitor T/S did not affect the expression of genes utilizing folate in the purine and methionine biosynthesis pathways, indicating that there is no signal transduction pathway connecting folate starvation to the transcriptional regulation of these pathways.

In addition, although MEM did not efficiently kill the high inoculum of bacteria at any concentration (Fig. S3a), transcriptional changes upon MEM treatment were observed in CA-MHB and M9 (Fig. S4c). These observations highlight the complementary nature of omics measurements to standard AST and killing assays, since they can describe differential responses that are not immediately clear from AST alone.

Taken together, we have presented an analytical pipeline for probing and contextualizing the transcriptomic effects of antibiotic treatment using a model organism. Although our analysis was focused on subinhibitory effects of antibiotics, this approach could be extended to investigate inhibitory or lethal doses of antibiotics. As publicly available transcriptomic data sets grow rapidly in size and diversity (52), this pipeline can be applied to pathogenic strains and organisms grown in more complex physiological media, e.g., human serum. Future studies could explore the metabolic and transcriptional shifts associated with antibiotic treatment, leading to a “white-box” approach that connects antibiotic-treated transcriptomes to biochemical knowledge (9).

## MATERIALS AND METHODS

### Bacterial strains and growth conditions.

E. coli strain MG1655 was grown in three media: (i) M9 minimal medium (47.8 mM Na_2_HPO_4_, 22 mM KH_2_PO_4_, 8.6 mM NaCl, 18.7 mM NH_4_Cl, 2 mM MgSO_4_, and 0.1 mM CaCl_2_) supplemented with 0.2% (wt/vol) glucose (M9); (ii) Roswell Park Memorial Institute 1640 (RPMI; Thermo Fisher Scientific) supplemented with 10% LB (R10LB); and (iii) Mueller-Hinton broth (Sigma-Aldrich) supplemented with 25 mg/liter Ca^2+^ and 12.5 mg/liter Mg^2+^ (CA-MHB). Glycerol stocks of E. coli were streaked onto Luria broth agar (LB agar) and grown at 37°C overnight.

### Antibiotic susceptibility testing.

Antibiotic susceptibility was determined as described previously (6). Briefly, E. coli-streaked plates were used to inoculate overnight cultures in the desired media. E. coli overnight cultures were used to inoculate pre-experimental cultures the following day. Pre-experimental cultures were grown to the midlogarithmic phase (OD_600_^ ^= ∼0.4) and then diluted to an OD_600_ of 0.002 (∼1 × 10^5^ CFU/ml) to create the experimental cultures. Concentrated antibiotic stocks were serially diluted in 1× DPBS (Corning) to create 10× stocks of each antibiotic at the indicated concentrations. Then, 180-μl portions of the experimental cultures were combined with 20 μl of the desired 10× antibiotic stock in Costar flat-bottom 96-well plates (Corning, catalog no. 3370) and incubated with shaking at 200 rpm at 37°C overnight. Bacterial growth (OD_600_) was determined approximately 20 h later utilizing a Enspire Alpha multimode plate reader (Perkin-Elmer). To calculate the MIC_90_, defined as the amount of drug required to inhibit ≥90% of the growth of the untreated controls, the density of each drug-treated well was compared to untreated control. MICs for high-density cultures were performed as described above, except with a starting OD_600_^ ^of  ∼0.05 after dilution.

### Killing assays.

Mid-log-phase E. coli MG1655 was used to inoculate experimental cultures of either glucose M9, R10LB, or CA-MHB with approximately 2.5^ ^× 10^7^ CFU/ml (OD_600_^ ^= ∼0.05). Antibiotics were added, and cultures were incubated as described for the antibiotic susceptibility assays. After 20 h, plates were removed from the incubator and wells were serially diluted 10-fold in their respective media from 10^0^ to 10^−7^. Then, 20 μl of each serial dilution was spot plated onto LB agar, followed by incubation at 37°C overnight to enumerate the CFU.

### RNA-seq expression profiling and processing.

Total RNA was sampled from duplicate cultures. Strains were cultured overnight in the respective media as described above. Mid-log-phase cultures were diluted (starting OD_600_ of ∼0.05) into 30 ml of medium for untreated conditions or 30 ml of medium containing the MIC_90_ of the respective antibiotic for the respective media (Table 1). Flasks were incubated for 30 min at 37°C with shaking. Cell broth was centrifuged, and the supernatant was removed. RNA extraction and library preparation were performed as described previously (12). Raw sequencing reads were performed as described previously (12). Differential expression was performed using DESeq2 (53), with a log_2_-fold change cutoff of 1.5 and *q* value cutoff of 0.05.

### ICA.

Log-transformed transcripts per million (log-TPM) expression levels were concatenated to the PRECISE data set. ICA and iModulon processing were performed as described previously (12). Briefly, we executed FastICA 100 times with random seeds and a convergence tolerance of 10^−7^. We constrained the number of independent components (ICs) in each iteration to the number of components that reconstruct 99% of the variance as calculated by PCA. The resulting ICs were clustered using DBSCAN to identify robust ICs, with the following parameters: an epsilon value of 0.1 and a minimum cluster seed size of 50. This process was repeated 10 times, and only ICs that consistently occurred in all runs were kept.

As described in Sastry et al. (12), iModulons were extracted from ICs by iteratively removing genes with the largest absolute value and computing the D’Agostino K^2^ test statistic of the resulting distribution. Once the test statistic fell below a cutoff, which was identified through a sensitivity analysis (12), we designated the removed genes as the “iModulons.”

### Gene set enrichment analysis.

To associate iModulons to regulators, we compared the set of significant genes in each iModulon to each regulon (defined as the set of genes regulated by any given regulator) using the two-sided Fisher exact test (FDR < 10^−5^), as described by Sastry et al. (12). In addition, combined regulon enrichments were calculated to identify joint regulation of iModulons (such as Fnr+NarL), using both intersection (+) and union (/) of up to three regulons. Final iModulon-regulator associations were determined through manual curation of enriched regulators.

Gene set enrichment analysis for differentially expressed genes was performed as described above, using only single regulator enrichments (FDR < 0.01).

### Explained variance.

Explained variance between two conditions was calculated as follows:

$$\text{Explained}\;\text{Variance}{}_{k}\;=\frac{\;\Sigma {\left(\Delta X\right)}^{2}\;-\;\;\Sigma {\left(\Delta X\;-\;S_{k}\Delta A_{k}\right)}^{2}}{\Sigma {\left(\Delta X\right)}^{2}\;},$$

where *k* is the iModulon of interest. The total explained variance was calculated similarly:

$$\text{Cumulative}\;\text{Explained}\;\text{Variance}=\frac{\;\Sigma {\left(\Delta X\right)}^{2}\;-\;\Sigma {\left(\Delta X\;-\;S\Delta A\right)}^{2}}{\Sigma {\left(\Delta X\right)}^{2}\;}.$$

The difference between the CEV and the sum of the explained variance is the confounding variance.

### Differential iModulon activity.

Differentially activated iModulons were computed with a similar process as previously detailed (12). For each iModulon, the average activity of the iModulon between biological replicates, if available, was computed. The absolute value of the difference in iModulon activities between the two conditions was then compared to the fitted log-normal distribution of all differences in activity for the iModulon. iModulons that had an absolute value of activity greater than 7 and an FDR below 0.1 were considered to be significant. The number of DIMAs was computed for each unique pair of conditions in the PRECISE 2.0 compendium.

### Oxygen consumption assay.

The oxygen consumption assay was performed using Abcam’s extracellular oxygen consumption assay kit (ab197243). Assay was performed as per the manufacturer’s suggested protocol. Exponentially growing bacterial cultures were diluted to an OD_600_ of ∼0.1, and 100 μl of the diluted cultures was added to a flat and transparent bottom 96-well sterile half-area cell culture plate. Dye was added as suggested in the manufacturer’s protocol, and wells were sealed with mineral oil. The time-resolved fluorescence was recorded in a kinetic cycle with 2 min interval with a 30-μs excitation-emission time lag and a 100-μs integration time using a Tecan infinite 200Pro. Plates were incubated at 37°C in a shaking mode (2-mm orbital amplitude). The OD_600_ was recorded in parallel to normalize for the cell number. Three independent replicates were used for every condition.

### Data availability.

All raw data are available on NCBI Gene Expression Omnibus (GEO) under accession number GSE159494. Processed data are available in the supplemental material. The code to compute iModulons is publicly available on Github at https://github.com/SBRG/precise-db.

10.1128/mSphere.00443-21.1 DATA SET S1 Expression levels, iModulon gene weights, and iModulon activities. Download Data Set S1, XLSX file, 18.5 MB. Copyright © 2021 Sastry et al. 2021 Sastry et al. https://creativecommons.org/licenses/by/4.0/ This content is distributed under the terms of the Creative Commons Attribution 4.0 International license.
