# Supplementary figures and images for: Machine Learning of Bacterial Transcriptomes Reveals Responses Underlying Differential Antibiotic Susceptibility
Source: mSphere. 2021 Aug 25;6(4):e00443-21. doi: 10.1128/mSphere.00443-21 (PMC8386450; doi:10.1128/mSphere.00443-21)

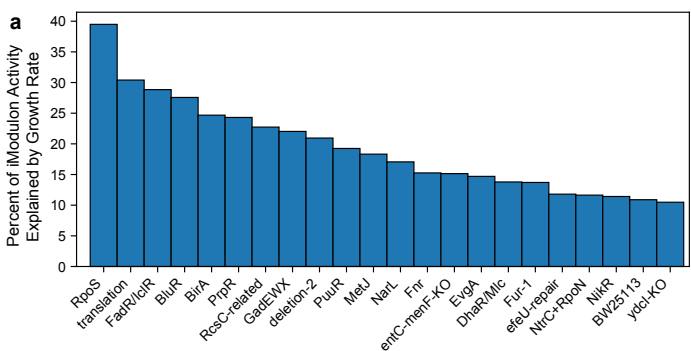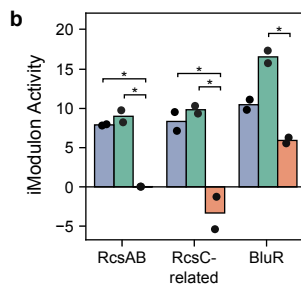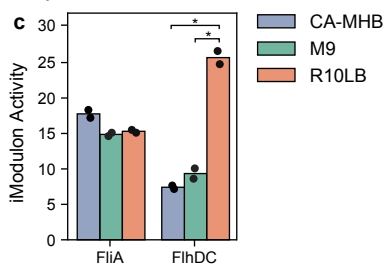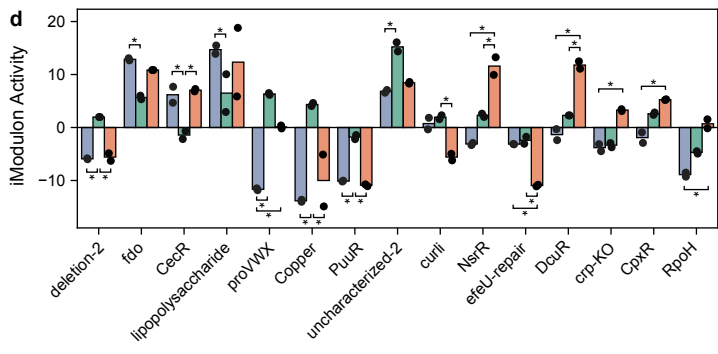

Supplement: FIG S2 [file msphere.00443-21-sf002.pdf]

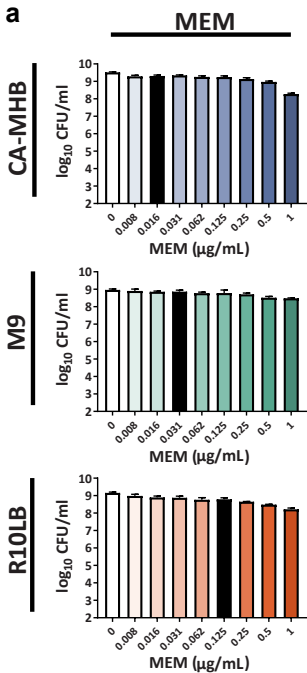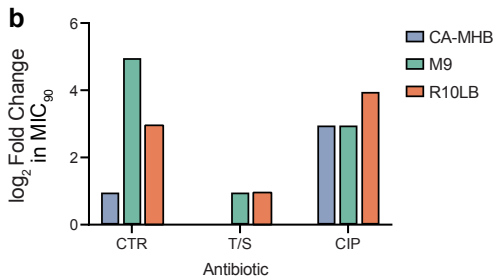

Supplement: FIG S3 [file msphere.00443-21-sf003.pdf]

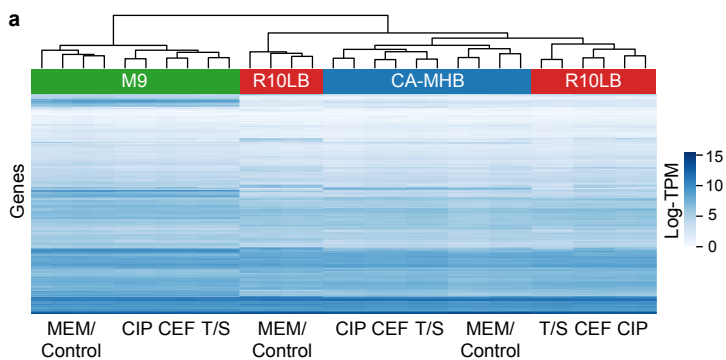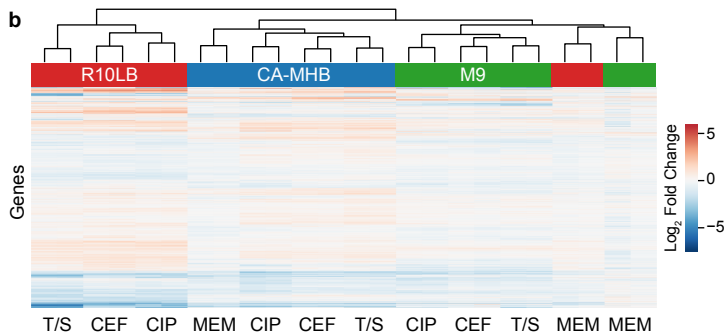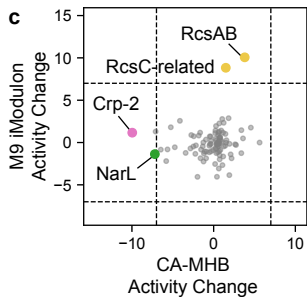

Supplement: FIG S4 [file msphere.00443-21-sf004.pdf]

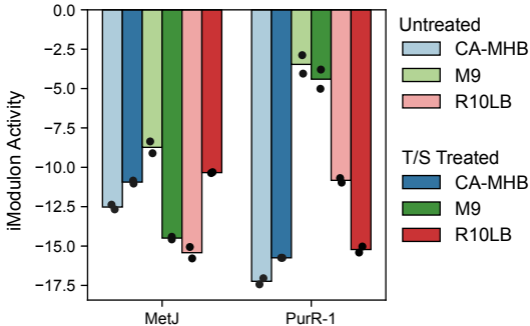

Supplement: FIG S5 [file msphere.00443-21-sf005.pdf]
