# Supplementary material for: Machine Learning of Bacterial Transcriptomes Reveals Responses Underlying Differential Antibiotic Susceptibility
Source: mSphere. 2021 Aug 25;6(4):e00443-21. doi: 10.1128/mSphere.00443-21 (PMC8386450; doi:10.1128/mSphere.00443-21)
Supplement: FIG S1 [file msphere.00443-21-sf001.pdf]

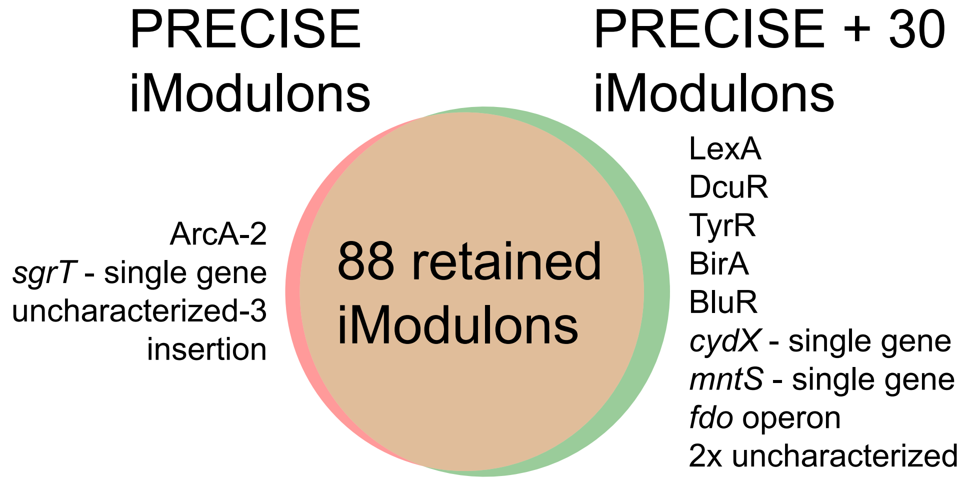
PRECISE
iModulons
PRECISE + 30
iModulons
ArcA-2
sgrT - single gene
uncharacterized-3
insertion
88 retained
iModulons
LexA
DcuR
TyrR
BirA
BluR
cydX - single gene
mntS - single gene
fdo operon
2x uncharacterized
