# Supplementary material for: Machine Learning of Bacterial Transcriptomes Reveals Responses Underlying Differential Antibiotic Susceptibility
Source: mSphere. 2021 Aug 25;6(4):e00443-21. doi: 10.1128/mSphere.00443-21 (PMC8386450; doi:10.1128/mSphere.00443-21)
Supplement: FIG S6 [file msphere.00443-21-sf006.pdf]

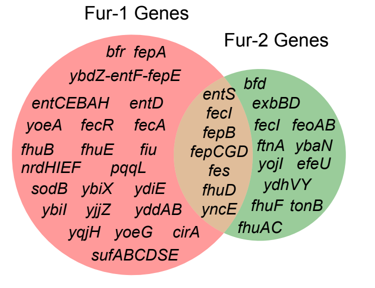

Fur-1 Genes
Fur-2 Genes
bfr fepA
ybdZ-entF-fepE
entCEBAH entD
yoeA fecR fecA
fhuB fhuE fiu
nrdHIEF pqqL
sodB ybiX ydiE
ybiI yjjZ yddAB
yqjH yoeG cirA
sufABCDSE
entS
fecI
fepB
fepCGD
fes
fhuD
yncE
bfd
exbBD
fecI feoAB
ftnA ybaN
yojI efeU
ydhVY
fhuF tonB
fhuAC
